# Measuring and Interpreting Nuclear Transport in Neurodegenerative Disease—The Example of C9orf72 ALS

**DOI:** 10.3390/ijms22179217

**Published:** 2021-08-26

**Authors:** Marije F. W. Semmelink, Anton Steen, Liesbeth M. Veenhoff

**Affiliations:** European Research Institute for the Biology of Ageing, University of Groningen, University Medical Center Groningen, 9713 AV Groningen, The Netherlands; m.f.w.semmelink@rug.nl (M.F.W.S.); a.steen@umcg.nl (A.S.)

**Keywords:** nuclear transport, nuclear pore complex, nuclear transport receptor, C9orf72, amyotrophic lateral sclerosis (ALS)

## Abstract

Transport from and into the nucleus is essential to all eukaryotic life and occurs through the nuclear pore complex (NPC). There are a multitude of data supporting a role for nuclear transport in neurodegenerative diseases, but actual transport assays in disease models have provided diverse outcomes. In this review, we summarize how nuclear transport works, which transport assays are available, and what matters complicate the interpretation of their results. Taking a specific type of ALS caused by mutations in *C9orf72* as an example, we illustrate these complications, and discuss how the current data do not firmly answer whether the kinetics of nucleocytoplasmic transport are altered. Answering this open question has far-reaching implications, because a positive answer would imply that widespread mislocalization of proteins occurs, far beyond the reported mislocalization of transport reporters, and specific proteins such as FUS, or TDP43, and thus presents a challenge for future research.

## 1. Introduction

### 1.1. Nucleocytoplasmic Transport

**Figure 1:**
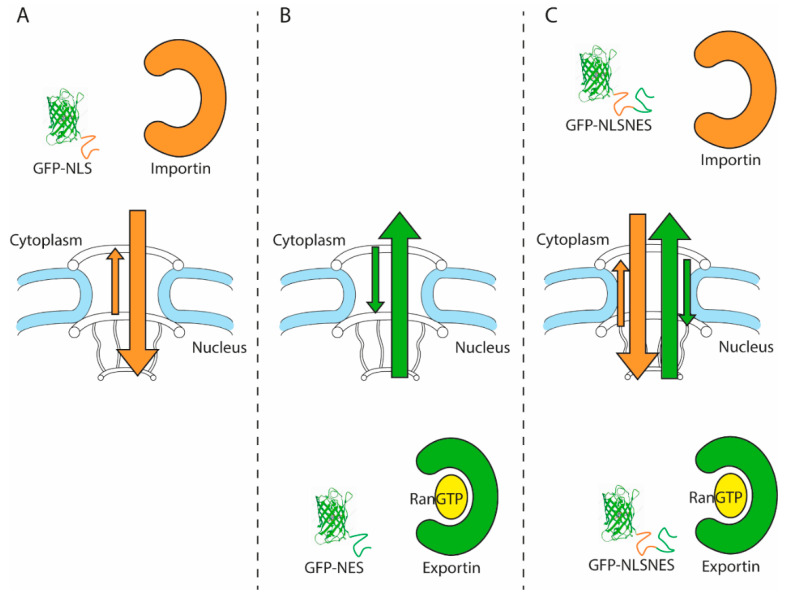
Nucleocytoplasmic transport can be measured with fluorophore-tagged transport reporters. (**A**) GFP tagged with an NLS accumulates in the nucleus owing to constant active import, which is faster than the passive leak in the opposite direction. (**B**) GFP tagged with an NES accumulates in the cytosol owing to constant active export, which overpowers passive influx. (**C**) GFP tagged with both an NLS and NES sequence accumulates depending on the strongest signal, as it is both actively imported and exported and passively diffuses across the nuclear pore. Passive diffusion (=influx and efflux) is dependent on the concentration gradient of the reporter, the number of NPCs, and their permeability.

Nucleocytoplasmic transport (NCT) occurs through nuclear pore complexes (NPCs), large protein complexes embedded in the nuclear membrane. NPCs facilitate and regulate the import and export of proteins and RNA between the nucleus and cytoplasm. Small proteins will rapidly diffuse through the NPC, but the passage of larger molecules is hindered by the NPC’s permeability barrier [1,2]. The association of proteins or RNAs with specific importins, exportins, and transportins, grouped together as nuclear transport receptors (NTRs), facilitates the passage of macromolecules, large and small, across the barrier (Figure 1).

The NPC is made up of proteins called nucleoporins (Nups) that build the cylindrical structure embedded in the nuclear membrane. FG-Nups containing phenylalanine-glycine (FG) repeats constitute roughly half of the mass of the NPCs [3] with about 200 copies of FG-Nups found in each pore [3,4]. The precise nature of the permeability barrier formed by the FG-Nups is still under debate, but what is clear is that the NTRs associate directly with the FG-Nups, even with specificity to certain Nups over others [5,6,7,8,9,10], to facilitate the passage of NTR–cargo complexes. Additionally, a pool of importin-β functions as a stable component of the NPC’s permeability barrier [11]. The differential localization of NPC-associated proteins in a single yeast nucleus suggests that NPCs may even have specialized transport functions [12].

In baker’s yeast, there are 19 known NTR [13,14,15,16], whereas in human cells, 30 NTRs [17] are identified. The substrate specificity of some, but not all NTRs has been mapped, and jointly they bind a large and diverse group of cargoes, while some redundancies exist [18]. The importin-β superfamily is the largest class of NTRs; they can either directly bind to their cargo, or via an adaptor protein, an importin-α isoform [19]. Exportins include CRM1, LOS1, and the MEX67-MTR2 (or human TAP/p15) complex for mRNAs and tRNAs [20], MSN5 for tRNAs, and CAS/CSE1 specifically exports Impα/Kap60 [21]. Of note, while general protein export is mediated only by the major exportin CRM1, many importins cooperate for general protein import. NTRs bind their cargoes through a nuclear localization signal (NLS) or a nuclear export signal (NES) encoded on a cargo molecule.

Ran, a member of the Ras family of small GTPases, is in its GTP bound state in the nucleus and its GDP bound state in the cytosol. This gradient functions to ensure that the cargoes are accumulated in the nucleus or cytosol by promoting the removal of cargo from importins and the loading of cargo to exportins [22,23,24]. Whereas importins bind to either the NLS of their cargo or to the RanGTP, exportins bind to both the NES of their cargo and the RanGTP cooperatively. Therefore, cargo release from importins depends on RanGTP binding, while cargo release from exportins needs RanGTP hydrolysis. RanGTP levels in the nucleus are maintained by the guanine nucleotide exchange factor RCC1 or RanGEF. RanGTP is converted to RanGDP in the cytoplasm by the GTPase-activating protein RanGAP. NCT is thus an interplay between the NPCs, with a number of different NTRs recognizing cargo through sorting signals and a gradient of Ran in its GTP or GDP bound state.

### 1.2. Amyotrophic Lateral Sclerosis (ALS) 

ALS is a neurodegenerative disease that affects neurons in the brain and spinal cord. Amyotrophy is the degeneration of spinal motor neurons, which leads to denervation and muscle wasting. Lateral sclerosis refers to the scarring (sclerosis) of the lateral axons, which control voluntary movement [25]. In most cases, the cause for ALS remains unknown, but 5–10% of ALS cases are caused by autosomal dominantly inherited mutations [26], of which almost 40% in Caucasians are in *C9orf72* [27]. Specifically, a G_4_C_2_ hexanucleotide is repeated up to thousands of times in C9-ALS patients [28,29], and translated via repeat-associated non-AUG (RAN) translation. This non-canonical initiation of translation allows elongation of a repeat sequence in the absence of an AUG starting codon, in multiple reading frames, thus leading to multiple dipeptide repeat proteins (DPRs) [30]. For *C9orf72*, the sense RNA encodes glycine-alanine (GA), glycine-proline (GP), and glycine-arginine (GR) repeat proteins. The antisense transcript produces proline-arginine (PR), glycine-proline (GP), and proline-alanine (PA) repeat proteins [31,32]. On a molecular level, C9-ALS disease is thus associated with multiple disease-related biomolecules, DNA, RNA, and DPRs. Hence, C9-ALS models introduce the G_4_C_2_ repeat DNA, or express or inject a single DPR of a certain length.

### 1.3. Nucleocytoplasmic Transport and C9orf72 ALS

A growing body of work suggests that changes in components of the nucleocytoplasmic transport machinery are related to neurodegenerative diseases (reviewed in [33,34,35,36,37,38,39,40,41,42,43]), but also specifically ALS. Firstly, the C9orf72 protein was found to interact with both importin-β1 and Ran-GTPase [44]. Moreover, effects on toxicity were reported; it was found that overexpression of RanGAP reduces toxicity of G_4_C_2_ repeats [45], downregulation of NTRs enhances G_4_C_2_ toxicity [46], and toxicity caused specifically by PR_50_—50 repeats of PR—is reduced by NTR overexpression [47]. An overview of NCT related genes that modify toxicity can be found in the accompanying review by Vanneste et al [48]. Furthermore, GA_50_ was reported to bind RanGAP1 in cytoplasmic inclusions [49], and poly-PR and -GR can directly bind to importin-β [50]. Moreover, the nuclear and cytoplasmic distributions of major protein components of NPCs were found to be altered in ALS [51]. Mislocalization or aggregation of NTRs has also been linked to C9-ALS (reviewed by Hutten and Dormann [39]) and mislocalization of NTRs and Nups has been shown in ALS post-mortem tissue and ALS models (reviewed in the accompanying review [48]. PR repeats are reported to directly bind to nuclear pores [52]. Further data linking altered NCT to C9-ALS include the following: nuclear RNA retention [46,52], mislocalization of Pom121 by poly-GA [49], reduced nuclear RCC1 (RanGEF) in *C9orf72*-carrier induced neurons [47], and increased cytoplasmic localization of neuronal RNA-binding protein Elav [53]. RanGAP was also found to be mislocalized in *C9orf72*-carrier induced neurons [47], but not in other models of *C9orf72*-associated neurodegeneration [47,54,55,56]. Thus, there is evidence from different model systems and experimental methods that nucleocytoplasmic transport components—NTRs, RanGAP, RanGEF, and Nups—are impacted in C9-ALS.

From the plethora of effects on NCT components, one would expect that the kinetics of NCT is impacted in C9-ALS. Establishing if the transport kinetics of NPCs in C9-ALS is altered is important, as a positive answer would suggest a common cause in ALS, i.e., mislocalization of many proteins, including ALS-related FUS and TDP43. A negative answer would suggest the NCT machinery is a robust system, and not causal to the phenotype of C9-ALS. This question has indeed been addressed in seven studies [45,50,52,55,57,58,59], which are summarized in Table 1; we included only those studies that make use of C9ALS models and mobile reporters. We did not include papers with full length proteins, as their localization may be impacted by other processes, such as retention. Table 1 shows the used reporters (i.e., usually a fluorescent protein with a specific NLS and/or NES sequence), the NTRs associated with the used NLS/NESs (importin-α/β complex, TNPO1 (Kapβ2), and CRM1), the C9-ALS model (i.e., introduction of repeat DNA, or expression or injection of a specific DPR), and the cell model. From a brief look at the joint data, it is not obvious if transport kinetics are altered in C9-ALS, as some experiments report changes, while others do not. To understand what one may conclude from the results from these diverse experimental setups and why there are seemingly inconsistent outcomes, we next discuss commonly used transport assays to then come back to the nuclear transport process in more detail to illustrate the complications in interpreting their outcomes.

### 1.4. Commonly Used Nuclear Transport Assays

The assays monitoring the kinetics of nuclear import and/or export generally use small inert reporters, such as GFP with a sorting signal, to control and limit reporter retention [60]. A GFP-NES or GFP-NLS reporter will passively pass the NPC down the concentration gradient owing to its small size, but additionally be actively transported owing to the NES/NLS [60]. Thus, its localization at steady state is the result of continuous nuclear import and efflux in the case of GFP-NLS, and export and influx in the case of GFP-NES (see Figure 1). The ratio between the fluorescence levels in the nucleus and cytosol provides the most common transport readout (the N/C ratio). The steady state localization of reporters encoding both an NLS and NES report on the ratio between import and export [61]. Provided that proper controls for the functionality of the NLS and NES are performed, this strategy has the advantage that, with a single cell line/model, one can monitor both import and export.

To measure actual rates of transport and influx/efflux, there are several strategies. Influx rates can be measured by monitoring the passive nuclear entry of larger cargoes, e.g., multimers of GFP, of proteinA, and of maltose-binding protein moieties [1,2]. The passive diffusion through the pores is affected by the overall size and shape of the protein, and its surface properties [62]. For example, surface histidines, cysteines, and arginines will facilitate the movement through the FG-Nup meshwork, and higher surface hydrophobicity increases the NPC passage [62]. Efflux and influx rates can also be measured using the GFP-NLS and GFP-NES reporters by inhibiting active import or export and following the subsequent loss of compartmentalisation. Import and export rates are derived from experiments where the import or export of GFP-NLS or GFP-NES reporters is temporarily disturbed, after which the re-establishment of the equilibrium can be quantified. Jointly, this collection of assays reports on the steady state ratios between nuclear entry and exit, or yields the individual rates of entry and exit by temporarily altering the steady state.

There are several options to block import or export. CRM1 export can be inhibited by Leptomicin B (LMB) [63], a fungicide that binds CRM1 irreversibly in the NES binding region, thus blocking the binding between CRM1 and its cargo [64,65]. A limitation of the use of LMB may be its off-target effects; it also inhibits the degradation of p53 and upregulates apoptosis in cancer lines [66], limits RNA translation [67], and leads to accumulation of poly(A)^+^ RNA within the nucleus [68]. Several import inhibitors are available that each block a specific transport route; e.g., importazole [69], ivermectin [70], and free importin-β-binding domain [50] inhibit the importin-α/β route, while the M9M peptide inhibits TNPO1 [71]. Alternatively, 2-deoxyglucose and/or sodium azide deplete cells from ATP, and can thus also be used to disrupt the RanGTP gradient. Depleting RanGTP inhibits major export and import pathways, but the different export routes are more sensitive to RanGTP depletion than the import via importin-α, importin-β, or transportin [72]. Especially in baker’s yeast, inhibition of import has been accomplished by anchor away strategies, such as anchoring of Kap95 to the plasma membrane ATPase using rapamycin inducible dimerization of FRB- and FKBP-domains attached to the NTR and the plasma membrane ATPase [73]. Methods that are performed at steady state and circumvent the use of drugs or poisons are based on fluorescence recovery after photobleaching (FRAP) and fluorescence loss in photobleaching (FLIP). In import studies, the nuclear area is bleached, and the recovery of nuclear fluorescence, which relies on the exchange of fluorescent molecules from the cytosol with bleached molecules from the nuclear compartments, is taken as a read-out for import rates [74,75]. As all assays, except those based on FRAP and FLIP, are subject to possible side effects of the interventions inhibiting transport, a combination of interventions is advisable.

Commonly used nucleocytoplasmic transport assays are performed in permeabilized cells, in live cells, or in in vitro assembled oocyte nuclei, and are listed in Table 2. A much used in vitro NCT assay uses digitonin permeabilized cells supplemented with exogenous recombinant transport factors [76]. This assay yields nuclei with functional NPCs while allowing to introduce the reporter proteins and NTRs at known concentrations and timepoints. The disadvantages are that permeabilization can relocate or extract soluble proteins [77] including transport factors and ATP [76], and potentially alter transport kinetics. In order to limit method artefacts, permeabilization assays are preferably combined with live-cell imaging [77]. The simplest transport measurements in live cells are those that determine the steady state distribution of GFP-NLS or GFP-NES reporters. Alternatively, as discussed above, the kinetics can be determined either based on the use of poisons to alter the steady state, or based on FRAP or FLIM. Lastly, like permeabilized cells, in vitro assembled oocyte nuclei also provide a system where reporter proteins and NTRs can be introduced at known concentrations and timepoints aiding kinetic measurements. Xenopus egg extracts contain the structural components of nuclei in a disassembled state, but will reconstitute nucleus-like structures around added DNA [78]. These nuclei actively exclude nonnuclear proteins and accumulate nucleoplasmin and proteins with NLSs [79,80,81], and import can be inhibited with lectin wheat germ agglutin, which binds to glycosylated FG-Nups [82]. Here, a combination of transport models may also provide the best assessment of the question at hand.

## 2. Nuclear Transport Up-Close: Complications when Interpreting Nuclear Transport Data

NCT assays rely on monitoring the localization of proteins to either the nuclear or cytosolic compartment (Figure 1). A change in localization can report a change in import or export efficiency by the joint action of NPCs, NTRs, and Ran. The interpretation of what drives the change in import or export efficiency and the contribution of each specific component (NPCs, NTRs or Ran), however, is often complicated for a number of reasons. 

First, cargoes are transported by dedicated transporters, which recognize their cargo via particular NLSs and/or NESs. However, often, a simple “one cargo–one NTR” relation does not exist, as specific NTR-isoforms show preference for certain NLSs, several NTRs can be redundant for one specific NLS, and a protein or protein complex can contain multiple localization sequences (see Table 3 for examples). NLS sequences are, among others, the classical NLS, which has a mono- or bipartite basic amino acid cluster [90]; the PY-NLS; and a RG-rich NLS [91]. Some importins have their own preferred sequences, such as yeast Kap121, which recognizes a non-conventional lycine-rich sequence [92]. There is not yet a full overview in which sequences are recognized by the importins, but attempts are ongoing to discover more NLSs [93]. The full complement of interactions between NTRs and cargoes in a specific cellular setting has not been established to date, hence a change in localization of one cargo cannot easily be connected to the activity of a specific NTR or NTR isoform.

Second, import rates depend on the availability of the NTRs [84,94,95]. The formation of a complex of NTR and cargo in the crowded cytosol is rate-limiting compared with the fast translocation through the NPC, which is reported to occur on the timescale of milliseconds for commonly used reporters [96]. Therefore, altering the concentration of NTRs impacts transport rates. For example, Kap121 and Kap123 recognize the same NLSs, and when expressed at similar concentrations, import rates are indistinguishable [84]. However, natively, the abundance of Kap123 is higher than that of Kap121, thus making Kap123-mediated import faster [84]. Import rates eventually saturate at high NTR levels, which must be owing to saturation of the NPCs, the RanGTP transport complex dissociation, or both [84]. The expression or availability of NTRs can change under stress conditions. For example, the importin Hikeshi, which uses ATP to import Hsp70 to limit shock-induced damage, is specifically upregulated under heat stress [16]. Moreover, conditions of oxidative stress and heat shock lead to rapid accumulation of Impα in the nucleus [97,98], and arsenite stress accumulates Impα in stress granules [99]. To explain a change in the localization of a reporter mechanistically, one would want to know whether the intervention, e.g., the introduction of DPRs or G_4_C_2_ DNA sequences, created a change in the levels of NTRs available for transport.

**Table 3:** Transport specificity and redundancy.

**I. Transport of Cargo Depends on a Localization Sequence that is Recognized by an NTR**	
NTR	NLS/NES
Impα/β	Sv40/cNLS: PKKKRKV [90] cMyc: PAAKRVKLD [100,101] Nucleoplasmin: KRPAATKKAGQAKKKK [102]
TNPO1	PY-NLS: R-X_2-5_-PY motif [103] e.g., FUS: RG-rich_46_RQDRRERPY [104]
Kap104	RG-rich motif [91]
Kap121	Lycine-rich sequence [92]
Attempts to discover more NLSs [93]	
CRM1	Φ-X_2-3_-Φ-x_2-_3-Φ-x-Φ; where Φ is I, M, F, V, and mostly L [105,106] PKI: e.g., MSLNELALKLAGLDI [58] HIV-1 Rev: LQLPPLERLTL
**II. Redundancy in NTRs for One Type of NLS**	
NTR1 NLSi-Cargo1 NTR2 NLSi-Cargo1	Different NTRs can recognize the same NLS, and thus transport the same cargo; e.g., Trn1, Trn2A, and Trn2B all bind the PY-NLS, and transport FUS [107]/HuR [108], as well as unique cargoes.
NTR1a NLSi-Cargo1 NTR1b NLSi-Cargo2	Different isoforms of one NTR may bind the same NLS, but transport other cargoes; e.g., importin-α isoforms bind the classical NLS, but, e.g., STAT1 is bound by α5, but not by α1 [109]. Importin-α3 only recognizes the NLS with appropriate N-terminal flanking residues [110].
**III. Redundancy in NTRs for One Type of Cargo**	
NTR1 NLSi -Cargo1 NTR2 NLSii-Cargo1	A group of cargoes, with distinct NLSs, can be recognized by different NTRs; e.g., Kap121 can substitute Kap123 for import of ribosomal proteins [111,112]
**IV. Combinations of Localization Sequences**	
NTR1 NLSi-NLSii-Cargo1 NTR2 NLSi-NLSii-Cargo1	One cargo may have two different NLSs; e.g., Nxf1 contains a cNLS for importin 4/11/α/β and a PY-NLS for Trn1/Trn2 [113,114,115,116,117].
NTR1 NLS-NES-Cargo1 NTR2 NLS-NES-Cargo1	One cargo may contain an NLS and an NES; cyclin B [118,119] and HIV Rev [105,120] contain an impβ1 NLS and a CRM1 NES.
NTR1 NES-adaptor Cargo1	Ribosomal pre60S particles bind to an adaptor protein (NMD3), which contains the NES [121,122].

Third, import rates also depend on the affinity between NTR and NLS, where over a certain range, a higher affinity will give a higher import rate [84,85,123]. However, a too high binding affinity prevents NTR–cargo complex dissociation and, consequently, inhibits transport [124,125]. Affinities can be temporarily altered by post-translational modifications (PTM), with phosphorylation of NTRs [126,127] and NLSs [128,129], the most studied PTM regulating NCT [130,131]. For example, transcription factors are only required for short periods of time and in small amounts, and sequences close to their NLS are phosphorylated to increase the affinity for importins [128,129] or cause retention in nucleus or cytoplasm [132]. However, methylation [133,134], ubiquitination [135,136,137], acetylation [138], and sumoylation [139] have also been shown to influence NTR binding. NTR binding can also be altered by physicochemical parameters, as is the case for ROS-induced formation of disulfide bonds between TRN1 and FOXO [140], which is reversed in the more reduced environment of the nucleus [141]. Proteolytic events can unmask NLS and NES sequences [142,143,144], whereas binding to another protein [145,146,147] or mRNA [148], or protein multimerization [149,150], can bury sorting sequences. To explain a change in the localization of a reporter mechanistically, one would want to know whether the affinity of the NTR for the NLS is altered. This is often not trivial to determine as the affinities determined with purified protein can be different from those determined in cytoplasmic extract [151] or those observed when multiple cargoes are presented simultaneously [152].

A fourth, and major, complication to interpret what drives the change in import or export efficiency is related to the fact that nuclear import of a specific heterologously expressed transport reporter (Figure 1) is always in competition with the transport of native cargoes. Because most NTRs have more than one specific cargo to transport, an increase in any of the cargoes of that NTR leads to lower N/C ratios of all its cargoes, including the reporter used to study transport [75,89]. Additionally, more cargo for one NTR can limit RanGTP availability to all NTRs, and slow down the recycling of importins and export. These aspects are easily overlooked when interpreting NCT data, but can lead to the false conclusion that the import kinetics by one specific NTR is reduced in a disease model compared with a wild type situation, while all that is changing is the concentration of competing cargo.

Lastly, it should be taken into account that changes in the localization of a reporter can be the result of changes to the NPCs themselves, both in number and in functionality. Recently, a disease-related reduced nuclear abundance of key Nups was shown in C9ALS and sporadic ALS iPSNs [153]. Theoretical considerations predict that fewer NPCs will support a higher accumulation of a GFP-NLS reporter in the nucleus [154], as the number of NPCs is likely limiting for efflux, but not for import [84,89]. Additionally, the deletion of one nucleoporin immediately translates to reduced export [155,156,157,158,159,160,161], but because the rate limiting step for import is receptor binding [95,154,162], cells still maintain high import rates even when FG-domains in the NPCs are deleted [6,163]. Specific NTRs preferentially bind specific FG-Nups, hence the presence or absence of certain FG-Nups may affect only specific import routes [6,164,165]. PTM has been reported to change the structure of pores. For example, in yeast, phosphorylation of Nup53 releases its binding partner Nup170, thus exposing binding sites for Kap121 on Nup170, limiting Kap121 import directly [166], a process that may also underlie reduced transport by TRN-1 and Kapβ [127]. Knowing the number and functionality of individual NPCs is a major challenge, and together with the previously mentioned complications of mapping the full complement of NTRS and cognate cargoes in a cell, these aspects provide the largest challenge in interpreting what underlies a change in the localization of a NCT reporter.

**Figure 2:**
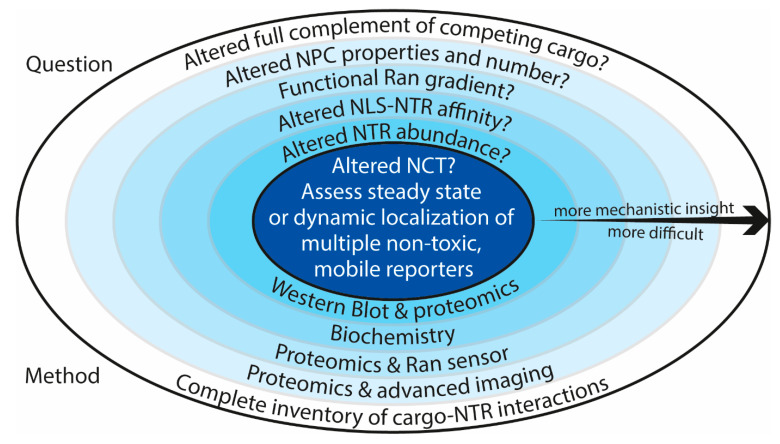
An overview of the questions related to answering whether nucleocytoplasmic transport is altered in a disease model; questions are shown in the top half of the image and methods are shown in the bottom half. The centre circle represents the main question of whether or not NCT is altered; this question can be answered by comparing the localization of transport reporters to either the nuclear or cytosolic compartment in wild type and disease models. The reporters have to be non-toxic, mobile, and preferably multiple NLS- or NES-containing reporters representing different transport pathways are used. To answer how NCT is altered, different additional questions have to be answered represented in layers of increasing complexity. In the case of the outermost layers, we enter into what is presently still largely uncharted scientific territory.

To summarize, in order to assess if the transport of proteins is altered in a disease model, well controlled steady state, or dynamic measurements of the location of mobile, non-toxic reporter proteins suffice. Preferably, multiple NLS- or NES-containing reporters representing different transport pathways are used. To understand what drives a possible change in the transport of these reporters, several additional, and rather complex, questions have to be addressed (summarized in Figure 2). In case of the outermost layers, we enter into what is presently still largely uncharted scientific territory.

All considerations above apply when comparing data obtained using different model systems. Nucleoporin expression changes between different cell types, tissues, and disease-states [167,168,169,170,171], as well as during development and ageing [172,173]. Moreover, the abundance of the NTRs differs between cell types. For example, Kapα levels differ between leukaemic cell lines [151] and between testis, ovary, spleen, heart, kidney, brain, or muscle cells [152,174,175,176,177]. Further, whereas TRN2A is expressed in HeLa cells, it is undetectable in HEK293T cells [108]. Kapα2 [178,179,180,181,182] and CRM1 [182,183,184,185,186,187,188,189] have been shown to be upregulated in different types of cancers, and NTR expression is regulated during development [190,191] and differentiation [192,193]. Thus, there is cell type variability of NPCs and cognate nuclear transport factors, which complicates the comparison of results from different studies.

For the studies related to C9-ALS, a further complication in the comparison of results is related to the fact that the DPRs impact cell physiology variably in the used biological systems as they localize differently and are toxic to different extents. In Table 4, we summarize the localization of different DPRs in different model systems and highlight whether they convey growth defects (indicated in different colours). For example, poly-PR is generally the most toxic DPR and PA is often non-toxic; this is the case in iPSC neurons [194], *S. cerevisiae* [47], rat neurons [194], mouse neurons, and HEK293 cells [195]. Yet, in zebrafish embryos, PR is less toxic than GR and equally toxic to PA [196]. PR is rarely found in patient material, usually attributed to its high toxicity, but if it is detected, it is found in aggregates in the cytoplasm [197,198]. This localization is not observed in most model systems where PR is nuclear or nucleolar [194,195,199,200,201,202]. The reports on poly-GA are diverse, reporting non-toxic [46,47,53,194,195,196,199,200,203,204] as well as toxic [49,200,205,206] effects. Interestingly, it seems that the toxicity of DRPs increases when cells are further differentiated into neurons [207]. Thus, even independent of the discussed cell type specific differences in the nuclear pores and cognate transport factors, the effect of DPRs on transport in the different C9-ALS models may be variable owing to differences in localization and toxicity.

## 3. Is Nuclear Transport Altered in C9-ALS?

The seven papers on NCT in C9-ALS [45,50,52,55,57,58,59] jointly report on 21 experiments assessing the localization of transport reporters in different C9-ALS models (Table 1). Clearly, despite obtaining solid and significant data, it is still difficult to conclude whether the data unambiguously establish that actual effects on NCT kinetics are observed when introducing C9-ALS-related peptides or DNA. How can six papers convincingly show alterations in the localization of transport reporters [45,50,52,55,57,59], whereas the seventh extensively shows no effects can be observed [58]?

Cell type-specific characteristics. When discussing several complications in the interpretation of NCT data, we mentioned cell type-specific transport characteristics, such as distinct NLS–NTR affinities or NTR abundances. This complication is clear when comparing the outcomes of the experiments in B and D (Table 1, for all further references in this section, see Table 1); the same reporter that is excluded from the nucleus in wild type motor neurons localizes to the nucleus in wild type *Drosophila* salivary glands. The same is observed comparing wild type HeLa Kyoto cells (L) and neuronal cells (N), where the reporter localizes differently in both cell types. These differences depending on cell type are also observed when specific DPRs are introduced. Namely, PR_20_ peptide injection reduces importin-α/β import in U2OS cells (F), but not in HeLa Kyoto cells (J). Similarly, poly-GA impacts the transport of an NLS_Sv40_-mNeonGreen_2x_-NES_PKI_ reporter in neuronal cells (N), but not in HeLa Kyoto cells (L). There may even be subpopulations within a cell type that behave differently. For example, when a subpopulation of cells with stress granules is selected, transport alterations are observed for (L), whereas this was not seen in the whole population [58]. This may be linked to the fact that arginine-rich DPRs can undergo liquid–liquid phase separation, and induce phase separation of a large set of proteins involved in RNA and stress granule metabolism [208]. Jointly, the cell type-specific localization of a reporter in wild type cells illustrates that cell type-specific differences in, among others, NPCs and cognate transport factors impact the transport assays (Table 1, compare L–N, B–D). In addition, the introduction of C9-ALS models adds to the variability between cell types (Table 1, compare F–J, N–L), possibly through the mentioned differences in localization and toxicity (Table 4).

Sorting signals. When discussing the different transport assays available, their possible technical complications were mentioned. A potential issue when using reporters with multiple localization signals relates to the fact that GFP has an intrinsic tendency to accumulate in the nuclear compartment [62,209], irrespective of the presence of an NLS. Nuclear localization of a GFP-based reporter may thus not be driven by import, but rather by influx. For example, the data suggest that the Sv40NLS is not very effective in motor neurons, as it does not promote nuclear accumulation when the NES is removed (E). Without controls, we do not know whether the nuclear accumulation of a reporter is due to import or influx, and the impact of DPRs thus might be on influx rather than active transport. Determining the effect is complicated, as Hayes et al. show that passive diffusion of GFP and small fluorescent dextrans into the nucleus, and thus influx of molecules is increased by PR_10_ and GR_10_ [50]. Validating the functionality of the sorting signals in reporters with both NLS and NES sequences is thus important to prevent changes in influx being misinterpreted as changes in import.

Transport assays. Changes in transport induced by the introduction of C9-ALS models are quantified in different ways, reporting either the nuclear fluorescence or the cytosolic fluorescence (A–G) or a ratio relative to the total (H–N) or cytosolic (O–P) fluorescence. Measuring the steady state fluorescence in the nucleus relative to the cytosol or total fluorescence is less sensitive to changes in expression level than other readouts such as nuclear (or cytosol) fluorescence alone. This may have impacted the interpretation in this case as poly-PR (and poly-GR) can limit translation [58,204,210]. Hence, it is possible that the expression of the reporter is reduced in the C9-ALS-models (B,C), which caused a lower nuclear fluorescence, rather than an effect on import.

Mobile reporters are the basis of all transport assays, and a quantification of the mobile fraction in FRAP experiments can be used to confirm that they are indeed mobile. However, it is the rate of recovery after bleaching in the nuclear or cytosolic compartment that is a measure for the kinetics of transport. The experiment in (A), for example, measures the change in mobile fraction, rather than actual transport kinetics.

Regarding the choice of transport assays (Table 2) and reporter, we mentioned that it is preferable to assess different transport reporters when studying NCT, as different NTRs each represent unique import pathways with unique parameters—affinities for FG-Nups, expression levels, and competing cargoes—defining their import kinetics. This is illustrated in (H,I) where the localization of a TNPO1 cargo is insensitive to GA_149_, while that of an importin-α/β is sensitive. Moreover, each transport assay has advantages and disadvantages (Table 2), and even though cell permeabilization assays (G) and injections (F,G,J,K) are used frequently in the field, it would be helpful to combine permeabilized studies with live-cell imaging.

Jointly, in the case of these C9-ALS studies, several technical limitations may have impacted the interpretation of the data summarized in Table 1. Hence, at present, it is not firmly established whether the introduction of disease-related biomolecules, G4C2-DNA, RNA, and DPRs, impacts the transport kinetics by NPCs.

Thus, even though convincing data have been obtained confirming a relation to the NCT machinery in C9-ALS in general, it is difficult to conclude from the current transport data if and how C9-ALS affects the actual kinetics of NCT. For future studies, the choice of model systems is critical, both from the perspective of the diverse behaviour of C9-ALS related DPRs (Table 4) and the diversity in the characteristics of NCT in different cells types. Moreover, to facilitate the interpretation of transport assays mechanistically, one would ideally quantify all nucleocytoplasmic transport components (NTR abundance, NPC number and functionality, and the integrity of the Ran gradient), study different NTRs, and combine different transport assays. These in-depth studies are required to answer whether NCT is changed in C9-ALS, and potentially many proteins are mislocalized, leading to cellular alterations causing the disease, or, alternatively, that the NCT machinery is a robust system, and widespread mislocalization of nuclear proteins should not be expected to occur or be causal to the phenotype of C9-ALS. The challenges described are not unique to the case of C9-ALS, and answering the question of how NCT is impacted in neurodegenerative diseases represents a major opportunity for scientists from the nuclear transport and neuroscience communities.

## Figures and Tables

**Table 1:** Transport studies of C9-ALS.

Summary of Experimental Setup and Results of Nuclear Transport Studies in C9-ALS Models					
Reporter (Localization in Wildtype)	NTR	Model	Effect of Indicated C9-ALS DNA Repeats or DPRs on Transport Reporter		Reference
			Changed	Unchanged	
**(A)** NLS_Sv40_-tdTomato-NES_Rev_ (nuclear)	Impα/β complex CRM1	(G_4_C_2_)_n_ expression in C9-ALS iPSNs	Recovery of nuclear fluorescence after FRAP is decreased		Zhang 2015 [45]
**(B)** NLS_Sv40_-NES_PKI_-GFP (nuclear)		(G_4_C_2_)_30_ expression in *Drosophila* salivary gland cells	Nuclear intensity is decreased		
**(C)** NLS_RanGAP_-ΔNES_PKI_-GFP (nuclear)					
**(D)** NLS_Sv40_-NES_PKI_-GFP (nuclear excluded)		(G_4_C_2_)_30_ expression in motor neurons	Nuclear exclusion is increased		
**(E)** NLS_Sv40_-ΔNES_PKI_-GFP (cellular diffuse)					
**(F)** GFP-NLS_Sv40_-NES_PKI_ (nuclear excluded)	Impα/β complex CRM1	PR_20_ peptide injection in U2OS cells	Nuclear intensity after export inhibition by Leptomycin B is decreased (PR_20_ dose-dependent)		Shi 2017 [52]
**(G)** BSA-NLS_(Sv40)n_ (nuclear)	Impα/β complex	DPR peptide injection in permeabilized HeLa cells	Nuclear intensity is decreased (PR_20_ dose-dependent)	Nuclear intensity is unchanged (PG_20_/PA_20_)	
**(H)** RFP-NLS_TDP_ (nuclear)	Impα/β complex	DPR expression in HeLa cells	Cytoplasmic intensity (C/T) is increased (more so with GA_149_-GFP, than with GA_149_-GFP-NLS)	Cytoplasmic intensity (C/T) is unchanged (GFP-GR_149_/PR_175_-GFP) †	Khosravi 2017 [57]
**(I)** RFP-NLS_PY (hnRNPA1)_ (nuclear)	TNPO1			Cytoplasmic intensity (C/T) is unchanged (GA_149_/GR_149_/PR_175_)	
NES_Rev_-tdTomato-NLS_Sv40_	Impα/β complex CRM1	C9-ALS fibroblasts	Nuclear accumulation (N/C) is decreased		Chou 2018 [55]
**(J)** NLS_Sv40_-mNeon Green_2x_-NES_PKI_ (cytoplasmic)	Impα/β complex CRM1	DPR peptide injection in HeLa Kyoto cells		Nuclear accumulation (N/total) after export inhibition by Leptomycin B is unchanged (PR_20_/GR_20_)	Vanneste 2019 [58]
**(K)** NLS_PY (FUS)_-mNeon Green_2x_-NES_PKI_ * (nuclear)	TNPO1 CRM1			Nuclear accumulation (N/total) after import inhibition by MGM9 is unchanged (PR_20_/GR_20_)	
**(L)** NLS_Sv40_-mNeon Green_2x_-NES_PKI_ (nuclear)	Impα/β complex CRM1	mCherry-DPR lentiviral expression in Hela Kyoto cells	†† Nuclear accumulation (N/total) after export inhibition by Leptomycin B is increased (PR_100_)	Nuclear accumulation (N/total) after export inhibition by Leptomycin B is unchanged (PA_100_ /GR_100_ /GA_100_)	
**(M)** NLS_c-myc_-GFP_2x_-NES_ikβ2_ (nuclear)				N/total unchanged (PA_100_/GA_100_/GR_100_/ PR_100_)	
**(N)** NLS_Sv40_-mNeon Green_2x_-NES_PKI_ (cytoplasmic)		mCherry-DPR lentiviral expression in SH-Sy5y neuronal cells and iPSC motor neurons	Nuclear accumulation (N/total) after export inhibition by Leptomycin B is decreased (GA_100_)	Nuclear accumulation (N/total) after export inhibition by Leptomycin B is unchanged (PA_100_ /GR_100_ /PR_100_)	
**(O)** IBB-domain_KPNA2_-FRET sensor (nuclear)	Impβ	DPR Peptide injection in permeabilized primary mouse cortical neurons	Nuclear accumulation (N/C) is decreased (dose-dependent and more so with PR_20_/GR_20_ than with PR_10_/GR_10_), and minimal decrease with GA_10_/PA_10_	Nuclear accumulation (N/C) is unchanged (GP_10_)	Hayes 2020 [50]
IBB-domain_KPNA2_-FRET sensor (nuclear)	Impβ	DPR Peptide injection in permeabilized HeLa cells	Nuclear accumulation (N/C) is decreased (more so with PR_20_/GR_20_ than with PR_10_/GR_10_) †††	Nuclear accumulation (N/C) is unchanged (PA_10_/GA_10_ /GP_10_)	
GST-GFP-NLS_Sv40_ (nuclear)	Impα/β complex		Nuclear accumulation (N/C) is decreased (more so with PR_20_/GR_20_ than with PR_10_/GR_10_)	Nuclear accumulation (N/C) is unchanged (PA_10_/GA_10_ /GP_10_)	
**(P)** YFP-M9_hnRNPA1_-CFP (nuclear)	TNPO1		Nuclear accumulation (N/C) is decreased (more so with PR_20_/GR_20_ than with PR_10_/GR_10_)	Nuclear accumulation (N/C) is unchanged (PA_10_/GA_10_ /GP_10_)	
GCR_2_-GFP_2_-MBP-NLS_SV40_ (nuclear ‡)	Impα/β complex	DPR peptide addition to medium HeLa cells	Nuclear accumulation (N/C) is decreased (GR_25_)		Hutten 2020 [59]
GCR_2_-GFP_2_-NLS_SV40_ (nuclear ‡)			Nuclear accumulation (N/C) is decreased (GR_25_) ‡‡		

* K to P mutation in the PKI-NES. † PR_175_-GFP was lower expressed than the GFP-GR_149_ and GA_149_-GFP. †† Reduced protein translation for GR_100_/PR_100_ expressing cells. ††† Passive diffusion of GFP and small fluorescent dextrans into the nuclei of permeabilized HeLa cells increased by PR_10_ and GR_10_, not by GP_10_, GA_10_, or PA_10_. ‡ Nuclear with dexamethasone. ‡‡ Nuclear accumulation and solubility of control GCR_2_-GFP_2_ reporter (lacking NLS) not altered.

**Table 2:** Benefits and drawbacks of commonly used transport assays.

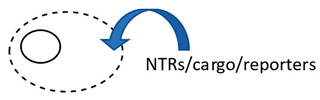	Cell permeabilization + Allows addition of specific concentration of cargo/NTR [76,83] - Permeabilization can relocate or extract soluble proteins [77] including transport factors/ATP [76]; might deplete NTRs from NPCs; and reduces concentrations of competitive cargo, which increases import rates [84]
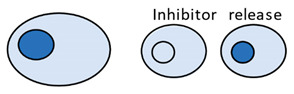	In vivo N/C ratios; use of inhibitors + N/C ratio is a robust read-out largely independent of reporter levels [85] - Expression/injection of reporter might be toxic; use of inhibitors to measure rates of nuclear entry and exit might be toxic
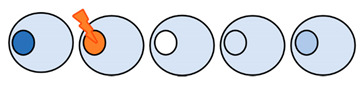	In vivo FRAP/FLIP + Direct measurement of kinetics of nuclear entry and exit; area under the curve indicates mobile fraction of the reporter - Expression/injection of reporter might be toxic; photobleaching induces ROS
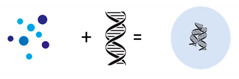	Xenopus egg extracts + Proteins can be added to/immunodepleted from the extract - No native cytosolic fraction ·Allows studying nuclear pore complex assembly, function [86], transport [87,88], and cargo competition [89].

**Table 4:** Toxicity and cellular localization of DPRs are not the same in all studied model organisms.

Cell Type	PR	PA	GP	GR	GA
Patients Frontal cortex	(Rare) Cytoplasmic aggregation [197,198]	(Rare) Cytoplasmic aggregation [198]	Cytoplasmic, paranucleolar aggregation [197]	Cytoplasmic aggregation [197]	Cytoplasmic, (paranuclear) inclusions [205]
				Paranucleolar aggregation [197]	Paranucleolar aggregation [197,205]
	(Rare) [198] Sparse: Cytoplasmic inclusions, or intra-nuclear inclusions [211]	Sparse: Cytoplasmic inclusions, cytoplasmic granular, or intranuclear inclusions [211]	Numerous: Cytoplasmic inclusions, cytoplasmic granular, or intranuclear inclusions [211]	(Rare) [198] Moderate: Cytoplasmic inclusions or intranuclear inclusions [211]	Numerous: Cytoplasmic inclusions, cytoplasmic granular, or intranuclear inclusions [211]
Patients spinal chord	Nuclear and extra-nuclear inclusions [194]				
iPSC neurons	Nucleolar [194] [194]			Cytoplasmic diffuse [194,203,212]	Perinuclear [212] or cytoplasmic inclusions [194,203]
Neuro2a cells	Cytoplasmic and nucleolar [213]	Cellular diffuse [213]	Cellular diffuse [213]	Cytoplasmically diffuse [213]	Cytosolic and nuclear inclusion bodies [213]
	[200]	[200]	[200]	[200]	[200]
Rat neurons	[194,214]	[194]	[194]	Length dependent toxicity [194]	[194]
				Nucleolar aggre-gation, cytoplas-mic diffuse [197,214]	Cytoplasmic aggregation [206]
Mouse neurons	Cytoplasmic diffuse plus inclusions [195,213]	Cellular diffuse [195,213]	Cellular diffuse [195,213]	Cellular diffuse [195,213]	Cytoplasmic aggregation [205,213]
					[195]
NCS34 cells (mouse motor neurons)	Nuclear aggregation [194,204,207]	Cytoplasmic aggregation [194,207]	Cellular diffuse [194,207]	Nuclear aggregation [194,204]	Cytoplasmic aggregation [194,204]
				[207]	
Differentiated SH-SY5Y cells (neuroblast)	Nuclear [202]	Diffuse cytosolic [202]		Diffuse cytoplasmic, plus nucleolar [202]	Stellate-like cytoplasmic inclusions [202]
HEK293 cells (cancer cells)	Cellular diffuse, nucleolar/cytoplasmic inclusions [205,206]	Cellular diffuse [205,206,213]	Cellular diffuse [205,206,213]	Cytoplasmic aggregation [206,213]	Cytoplasmic, occasionally nuclear aggregation [49,205,206]
	Cytoplasmic aggregates, plus nucleolar [213]			Nuclear aggregation [205]	
	Nucle(ol)ar [195]	Cellular diffuse [195]	Cellular diffuse [195]	Nucle(ol)ar [195]	Cellular [195,213]
HeLa cells (cancer cells)	Nucleolar [200]	Cellular diffuse [200]	Cellular diffuse, preferentially nuclear [200]	Nucleolar [200]	Paranuclear aggregation [200] Cytoplasmic inclusions [203]
				Cytoplasmic diffuse [203]	
				Cytoplasmic inclusions [213]	Diffuse cellular [213]
K562 cells (cancer cells)	[215]			[215]	
U2OS cells (cancer cells)	Nucleoli [201]			Nucleoli [201]	
CHO cells (Chinese ham-ster ovary)	[207]	[207]	[207]	[207]	
COS cells (fibroblast-like monkey kidney cells)			Cellular diffuse [213]	Cytoplasmic inclusions [213]	Cellular diffuse [213]
*C. elegans (muscle)*	Nucleolar [199]	Cellular diffuse, preferentially nuclear [199]		Cytoplasmic diffuse and nucleolar [199]	Cytoplasmic aggregation [199]
*D. melanogaster*	[53,193,199,202,211]	[53,194,200,216]	[46,200]	Cytoplasmic diffuse [203]	[46,53,194,200,203]
*S. cerevisiae*	[47]	[47]		[47]	[47]
Zebrafish embryo’s	[196]	[196]		[196]	[196]

Colour coded toxicity from red meaning highly toxic, intermediately toxic in orange, to green denoting non-toxic, as specified in each paper (not comparatively between model organisms). Reference to toxicity is in the right lower corner.

## Abbreviations

ALS	amyotrophic lateral sclerosis
C9-ALS	C9orf72-related ALS
DPRs	dipeptide repeat proteins
FG	phenylalanine glycine repeat
FLIP	fluorescence loss in photobleaching
FRAP	fluorescence recovery after photobleaching
FUS	fused in sarcoma
GA, GP, GR, PA, PR	glycine-alanine, glycine-proline, glycine-arginine, proline-alanine, proline-arginine repeats
LMB	leptomycin B
NES	nuclear export sequence
NLS	nuclear localization sequence
NP/NMP1	nucleophosmin
NPC	nuclear pore complex
NTR	nuclear transport receptor
Nups	nucleoporins
PKI	cAMP-dependent *protein kinase inhibitor*
PTM	post-translational modification
RAN translation	repeat-associated non-AUG translation
Ran	Ras-related nuclear protein
RanGAP	Ran GTPase activating protein
RanGEF	Ran guanine nucleotide exchange factorRBP RNA binding protein
ROS	reactive oxygen species
Sv40	simian virus 40
Rev	human immunodeficiency virus type 1 Rev protein
TDP-43	TAR-DNA binding protein 43
TNPO1	Kapβ2
TRN1	transportin 1
